# Inequality in the Frequency of the Open States Occurrence Depends on Single ^2^H/^1^H Replacement in DNA

**DOI:** 10.3390/molecules25163753

**Published:** 2020-08-18

**Authors:** Alexander Basov, Mikhail Drobotenko, Alexandr Svidlov, Eugeny Gerasimenko, Vadim Malyshko, Anna Elkina, Mikhail Baryshev, Stepan Dzhimak

**Affiliations:** 1 Kuban State Medical University, 350063 Krasnodar, Russia; son_sunytch79@mail.ru (A.B.); Intro-2@rambler.ru (V.M.); 2 Kuban State University, 350040 Krasnodar, Russia; mdrobotenko@mail.ru (M.D.); svidlov@mail.ru (A.S.); baryshev_mg@mail.ru (M.B.); jimack@mail.ru (S.D.); 3 Federal Research Center the Southern Scientific Center of the Russian Academy of Sciences, 344006 Rostov-on-Don, Russia; 4 Kuban State Technological University, 350042 Krasnodar, Russia; rosmaplus@gmail.com

**Keywords:** deuterium, DNA, mathematical model, open states, rotational movements of nitrogenous bases, dynamics of a double-stranded DNA molecule

## Abstract

In the present study, the effect of ^2^H/^1^H isotopic exchange in hydrogen bonds between nitrogenous base pairs on occurrence and open states zones dynamics is investigated. These processes are studied using mathematical modeling, taking into account the number of open states between base pairs. The calculations of the probability of occurrence of open states in different parts of the gene were done depending on the localization of the deuterium atom. The mathematical modeling study demonstrated significant inequality (dependent on single ^2^H/^1^H replacement in DNA) among three parts of the gene similar in length of the frequency of occurrence of the open states. In this paper, the new convenient approach of the analysis of the abnormal frequency of open states in different parts of the gene encoding interferon alpha 17 was presented, which took into account both rising and decreasing of them that allowed to make a prediction of the functional instability of the specific DNA regions. One advantage of the new algorithm is diminishing the number of both false positive and false negative results in data filtered by this approach compared to the pure fractile methods, such as deciles or quartiles.

## 1. Introduction

The deuterium concentration in the body plays an important role in the metabolic processes of living systems [1,2,3,4]. The biological effects caused by deuterium depleted water (DDW) have been studied at various levels: cellular [5,6,7,8], tissue [9], organismic [10,11,12,13,14]. It was found that low concentrations of deuterium in the drinking diet increase stress resistance in mammals [15], and may also have neuroprotective properties [16]. In addition, it was shown that a decrease in the deuterium content in the blood and brain of laboratory animals reduces the disruption of antioxidant enzymes functioning during hypoxia in comparison with animals that received water with a natural deuterium content [17]. The administration of water with a reduced deuterium content into the drinking diet leads to a change in the ^2^H/^1^H ratio in the body tissues due to the hydrogen isotopes exchange [18], in particular, as shown by the recent study, the antiproliferation effects of DDW are associated with the occurrence of an imbalance in the mitochondria between the production of reactive oxygen species and their neutralization [19]. Some studies have shown an increase in H_2_O_2_ production by mitochondria during their pre-incubation in the deuterium depleted media [20,21]. In addition, the mechanisms of DDW influence on biological systems from the point of view of the medical biochemistry are logically explained by a modification in the mitochondria energy balance [22,23].

Thus, to date, various biological effects caused by DDW have been examined in sufficient detail, and there is also an opinion that the main “fast” mechanism of the effect of low concentrations of deuterium in drinking water on the organism is a change in the parameters of the mitochondria work. However, it is important to understand the long-term effects of DDW administration, as well as its possible impact on DNA [24]. It is known that the deuterium bond energy is ~ 134 calorie /mol higher than the protium energy; therefore, deuterium forms a 5% stronger hydrogen bond [25]. The infiltration of a deuterium atom instead of protium into the hydrogen bond of the DNA double helix can cause a very noticeable temporary malfunction in the transmission of this information, most likely due to a delay in the opening of any hydrogen bond. Deuterium atoms get into the hydrogen bonds of DNA double helices because of the rapid protium-deuterium isotope exchange with the surrounding water molecules [26]. With a natural deuterium content of 156 ppm in water, the equilibrium probability of deuterium getting into each of the possible hydrogen bonds is small and equals approximately 2 × 10^−4^ [24]. In addition to fluctuation twists of the helix axis and rotations of adjacent pairs of nitrogen bases in the DNA molecule, the opening and closing of individual pairs of nitrogen bases occurs [27]. This process leads to changes in conformation and plays an important role in the reactions of DNA with chemical agents. Replacing a protium atom with deuterium affects the process of opening base pairs by increasing the energy required to break the bond. To date, the implementation of an experiment that would allow us to evaluate the effect of a deuterium atom on the processes of opening base pairs is difficult. A theoretical study of these processes is possible using methods of mathematical modeling, and one of the key conditions for the adequacy of the mathematical model of DNA is to take into account open states. Existing mathematical models of DNA are discussed in a number of reviews [27,28,29]. However, to describe the effects caused by the introduction of deuterium atoms to hydrogen bonding between base pairs, the upgraded Yakushevich model was chosen [30,31,32,33], which allows to take into consideration the energy of the hydrogen bonds between pairs of nitrogenous bases. Due to the introduction of an additional term, this model allows to consider the effects of dissipation caused by the viscosity of the medium surrounding the DNA molecule. In addition, in the model, due to the introduction of the torque constant of a given site of the sugar-phosphate chain, the interactions between adjacent base pairs are indirectly taken into account. Another argument in favor of choosing the Yakushevich mechanical model to describe the role of deuterium atoms in the opening and closing processes of base pairs was the fact that radial torsion models are able to take into account distortions of the DNA structure caused by external torsion stress. However, most physicochemical experiments are carried out on relaxed DNA, without such distortions. We can assume that the behavior of relaxed molecules is described equally well by radial and radial-torsion models [27].

In the presented work, in the framework of the mechanical DNA model, evaluation experiments were carried out on the effect of isotopic ^2^H/^1^H exchange in hydrogen bonds between pairs of nitrogen bases on the uneven probability distribution of open states along the gene length.

## 2. Methods

### 2.1. Mathematical Model

To simulate the processes of formation and dynamics of open states (OS) we will use a mathematical model that describes the rotational movement of nitrogen bases around the sugar-phosphate chain of a DNA molecule [34].

This mathematical model includes Newton’s equations:

(1′) $$I_{1}^{i}\frac{d^{2}\varphi_{1}^{i}\left(t\right)}{dt^{2}}=K_{1}^{i}\left[\varphi_{1}^{i-1}\left(t\right)-2\varphi_{1}^{i}\left(t\right)+\varphi_{1}^{i+1}\left(t\right)\right]\;-\delta^{i}\left(k_{12}^{i}R_{1}^{i}\left(R_{1}^{i}+R_{2}^{i}\right)\sin\varphi_{1}^{i}+k_{12}^{i}R_{1}^{i}R_{2}^{i}\sin\left(\varphi_{1}^{i}-\varphi_{2}^{i}\right)\right)\;+F_{1}^{i}\left(t\right),\;i=\bar{2,n-1},$$

(1″) $$I_{1}^{1}\frac{d^{2}\varphi_{1}^{1}\left(t\right)}{dt^{2}}=K_{1}^{1}\left[\varphi_{1}^{2}\left(t\right)-\varphi_{1}^{1}\left(t\right)\right]\;-\delta^{i}\left(k_{12}^{1}R_{1}^{1}\left(R_{1}^{1}+R_{2}^{1}\right)\sin\varphi_{1}^{1}+k_{12}^{1}R_{1}^{1}R_{2}^{i}\sin\left(\varphi_{1}^{1}-\varphi_{2}^{1}\right)\right)+F_{1}^{1}\left(t\right),$$

(1‴) $$I_{1}^{n}\frac{d^{2}\varphi_{1}^{n}\left(t\right)}{dt^{2}}=K_{1}^{n}\left[\varphi_{1}^{n-1}\left(t\right)-\varphi_{1}^{n}\left(t\right)\right]\;-\delta^{i}\left(k_{12}^{n}R_{1}^{n}\left(R_{1}^{n}+R_{2}^{n}\right)\sin\varphi_{1}^{n}+k_{12}^{n}R_{1}^{n}R_{2}^{n}\sin\left(\varphi_{1}^{n}-\varphi_{2}^{n}\right)\right)+F_{1}^{n}\left(t\right),$$

(2′) $$I_{2}^{i}\frac{d^{2}\varphi_{2}^{i}\left(t\right)}{dt^{2}}=K_{2}^{i}\left[\varphi_{2}^{i-1}\left(t\right)-2\varphi_{2}^{i}\left(t\right)+\varphi_{2}^{i+1}\left(t\right)\right]\;+\delta^{i}\left(k_{12}^{i}R_{2}^{i}\left(R_{1}^{i}+R_{2}^{i}\right)\sin\varphi_{2}^{i}-k_{12}^{i}R_{1}^{i}R_{2}^{i}\sin\left(\varphi_{2}^{i}-\varphi_{1}^{i}\right)\right)+F_{2}^{i}\left(t\right),\;i=\bar{2,n-1},$$

(2″) $$I_{2}^{1}\frac{d^{2}\varphi_{2}^{1}\left(t\right)}{dt^{2}}=K_{2}^{1}\left[\varphi_{2}^{2}\left(t\right)-\varphi_{2}^{1}\left(t\right)\right]\;+\delta^{i}\left(k_{12}^{1}R_{2}^{1}\left(R_{1}^{1}+R_{2}^{1}\right)\sin\varphi_{2}^{1}1-k_{12}^{1}R_{1}^{1}R_{2}^{1}\sin\left(\varphi_{2}^{1}-\varphi_{1}^{1}\right)\right)+F_{2}^{1}\left(t\right),$$

(2‴) $$I_{2}^{n}\frac{d^{2}\varphi_{2}^{n}\left(t\right)}{dt^{2}}=K_{2}^{n}\left[\varphi_{2}^{n-1}\left(t\right)-\varphi_{2}^{n}\left(t\right)\right]\;+\delta^{i}\left(k_{12}^{n}R_{2}^{n}\left(R_{1}^{n}+R_{2}^{n}\right)\sin\varphi_{2}^{n}-k_{12}^{n}R_{1}^{n}R_{2}^{n}\sin\left(\varphi_{2}^{n}-\varphi_{1}^{n}\right)\right)+F_{2}^{n}\left(t\right).$$

here:

$\varphi_{j}^{i}\left(t\right)$—is the angular deflection of the *i*-th nitrogen base of the *j*-th chain counted counterclockwise at time *t*;
$I_{j}^{i}$—is the rotational inertia of the *i*-th nitrogen base of the *j*-th chain;
$R_{j}^{i}$—is the distance between the center of inertia of the *i*-th nitrogen base of the *j*-th chain to sugar phosphate chain;
$K_{j}^{i}$—is the constant characterizing the torsion moment of the *i*-th segment of the *j*-th sugar phosphate chain;
$k_{12}^{i}$—is the constant characterizing the bond elastic properties of the *i*-th nitrogen base pairs;
$F_{j}^{i}\left(t\right)$—external influence on the *i*-th nitrogen base of the *j*-th chain at a time t,
n—is the number of nitrogene base pairs in the system.

The magnitude of the external force is taken as $F_{j}^{i}\left(t\right)=-\beta_{j}^{i}\frac{d\varphi_{j}^{i}}{dt}\left(t\right)+F_{0}\cos\omega t$, where summand $-\beta_{j}^{i}\frac{d\varphi_{j}^{i}}{dt}\left(t\right)$ models the effects of dissipation due to the interaction with water surrounding the DNA molecule, the term $F_{0}\cos\omega t$ is the external periodic impact.

In Equations (1′)–(2‴), the first term to the right of the sign of the equality describes the force acting on the *i*-th nitrogen base from the sugar-phosphate chain, the second term is the force from the complementary nitrogen base, and the third term is the external impact.

Thus, Equations (1′)–(2‴) allow us to simulate the hydrogen bond in the *i*-th pair ($\delta^{i}=1$, $k_{12}^{i}=k_{12}^{H,i}$), deuterium bond ($\delta^{i}=1$, $k_{12}^{i}=k_{12}^{D,i}$) and disruption of this bond ($\delta^{i}=0$). We will assume that the break between base pairs occurs if the potential binding energy in these pairs exceeds a certain critical value $E_{cr}^{H}$ for hydrogen bonds and $E_{\mathrm{cr}}^{D}$ for deuterium bonds, but if the potential energy in a pair with a broken bond is less than the critical value, then the bond is restored.

We add the initial conditions to Equations (1′)–(2‴):

(3′) $$\varphi_{1}^{i}\left(0\right)=\varphi_{1,0}^{i},\;\frac{d\varphi_{1}^{i}}{dt}\left(0\right)=\varphi_{1,1}^{i},$$

(3″) $$\varphi_{2}^{i}\left(0\right)=\varphi_{2,0}^{i},\;\frac{d\varphi_{2}^{i}}{dt}\left(0\right)=\varphi_{2,1}^{i},\;i=\bar{1,n}.\;$$

For definiteness, we’ll assume that at *t* = 0 the system is in equilibrium, i.e., in the initial conditions (3)

$$\varphi_{1,0}^{i}=\varphi_{1,1}^{i}=\varphi_{2,1}^{i}=0,\;\varphi_{2,0}^{i}=\pi ,\;i=\bar{1,n}.$$

Problem (1)–(3) is the Cauchy problem for a system of *2n* ordinary differential equations; in this work, all studies were carried out on the basis of a numerical solution of this system.

**Figure 1:**
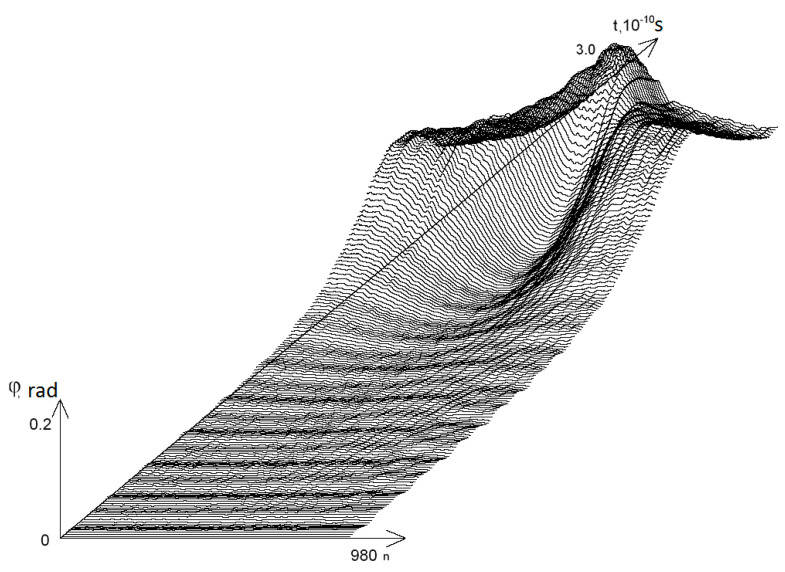
Graphs of angular deviations of the 1-st chain of DNA molecule nitrogenous bases over period of time: [0, t = 3.0 × 10^−10^ c].

On Figure 1 the graphs of the angular deviations of the 1-st chain of DNA molecule nitrogenous bases over period of time are presented: [0, *t* = 3.0 × 10^−10^ c].

### 2.2. The Effect of ^2^H/^1^H Exchange on the Probability of OS Formation

We will study the effect of ^2^H/^1^H exchange on the formation and dynamics of the OS using the example of a gene encoding interferon alpha 17. For this gene *n* = 980, the values of the Equations (1′)–(2‴) coefficients are shown in Table 1 (data taken from [30]), F_0_ = 0.526 × 10^−22^ J, ɷ = 0.4 × 10^12^ s^−1^.

We designate by P_0_ the probability of an OS formation in a molecule in which all pairs of nitrogenous bases are connected by hydrogen bonds; by P*_i_*, I = $\bar{1,n}$, the probability of an OS occurrence in a DNA molecule in which in the *i*-th nitrogen base pair one <any > hydrogen bond is replaced by deuterium.

The probabilities P_0_ и P*_i_*, I = $\bar{1,n}$, will be sought on the basis of a numerical solution of the problem (1)–(3). To do this, we will create a set of points *t_j_* = *j*Ʈ, *j* = $\bar{1,m}$, Ʈ = T/*m* in the segment [0, T]. Then, we will calculate at *t* = *t_j_* the ratio *q_j_* of the number of base pairs with a broken bond to the total number of base pairs *n*, then the value of P_k_ is equal to the arithmetic mean value over the points *t_j_* of these ratio:

$$P_{i}=m^{-1}\left(\overset{m}{\underset{j=1}{\sum }}q_{j}\right)$$

Since the deuterium bond is 5% stronger than hydrogen [25], we took $E_{cr}^{D}=k^{D}\cdot E_{cr}^{H}$, $k_{12}^{D,i}=k^{D}\cdot k_{12}^{H,i}$, *k^D^* = 1.05.

For various values of $E_{cr}^{H}$ values P_0_ и P*_i_*, *i* = $\bar{1,n}$ were calculated. The calculations were performed with accuracy 10^−6^ for T = 3.0 × 10^−10^ s, Ʈ = 0.0001 × 10^−10^ s.

To carry out calculations based on the mathematical model, a computer program was developed. It works as follows: when a deuterium atom enters hydrogen bonds between the first base pair of gene encoding interferon alpha 17,980 equations are solved (by the Runge–Kutta method of the 4th order), the average probability of occurrence of open states in the gene is calculated, then a similar calculation is performed for the second pair of bases, etc.

## 3. Results

**Figure 2:**
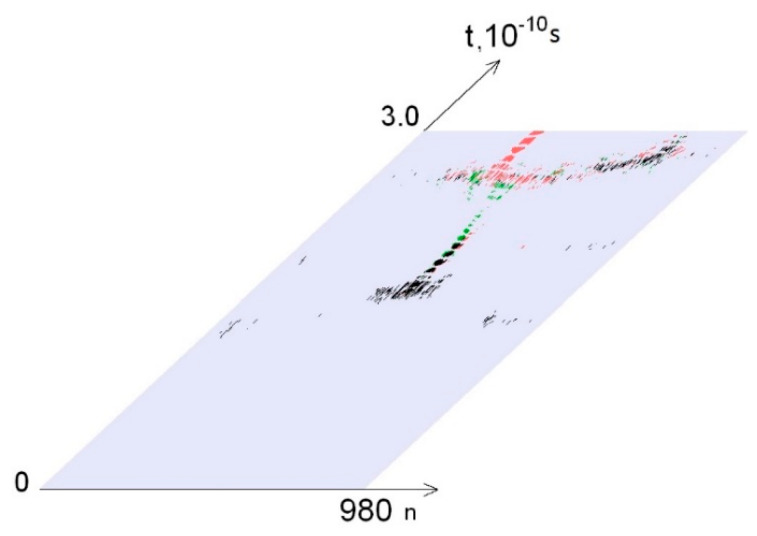
Dynamics of open states occurrence for the gene encoding interferon alpha 17, $E_{cr}^{H}$ = 0.31$\times 10^{-22}$ N·m. Note: green dots show open states dynamics, when deuterium was placed in *i*_min_ (365 base pair, Table 2), red dots show open states dynamics, when deuterium was placed in *i*_max_ (711 base pair, Table 2) and black dots represent coincidence values for *i*_min_ and *i*_max_.

The OS dynamics of the DNA molecule and the effect of ^2^H/^1^H exchange on it are illustrated on Figure 2, in which the zones of open states are highlighted in color. Moreover, Figure 2 corresponds to an energy $E_{cr}^{H}$ = 0.31 $\times \;10^{-22}$ N·m.

Table 2 contains data of the values of P_0_, the *i*_min_ and *i*_max_ numbers of nitrogen base pairs, the substitution of which in the hydrogen bond with deuterium bond leads to the lowest and highest values of the open state occurrence probability and the values of the $\;P_{i_{\min}}$ and $P_{i_{\max}}$.

Gene encoding interferon alpha 17 (IFNA17) and containing 980 nucleotide pairs were conditionally divided into three equal parts: I part (from the 1st to the 327th bases, *n* = 327), II part (from the 328th to the 653th bases, *n* = 326) and III part (from the 654th to the 980th bases, *n* = 327). In the whole gene we counted probabilities (P*_i_*) of occurrence of open states between different nitrogenous bases in double-stranded DNA dependent on the single ^2^H/^1^H replacement in base pair of each gene region. All these P*_i_* were arranged from P_*i*min_ to P_*i*max_ and each of them was compared to P_0_, which was determined as probability of OSs occurrence, when all hydrogen bonds in DNA are ^1^H. According to the serial number of each nucleotide base pair its affiliation to I part, II part or III part of IFNA17 was determined, and after that the amount of nucleobase pairs from the ranges “Maximum” and “Minimum” for the whole gene and its parts was calculated. Nitrogenous bases, which had P_*i*max_, and base pairs with the higher P*_i_* were selected as representatives of the maximum range (“Maximum”). Nitrogenous bases, which had P_*i*min_, and base pairs with the more-low P*_i_* were selected as representatives of the minimum range (“Minimum”). The ranges “Maximum” and “Minimum” were found by different below-presented methods: decile method [35], quartile method [35], and new method (Basov–Jimack algorithm, or BJ-algorithm).

- Decile method:
  (1) *i* ϵ range “Maximum” (top 10th decile, Dec_10_):
    P*_i_* ≥ P_*i*max_ − 1/10·(P_*i*max_ − P_*i*min_) ⇒ n_max_ = ƩnP_*i*_;
  (2) *i* ϵ range “Minimum” (bottom 1st decile, Dec_1_):
    P*_i_* ≤ P_*i*min_ + 1/10·(P_*i*max_ − P_*i*min_) ⇒ n_min_ = ƩnP*_i_*.
- Quartile method*:
  (1) *i* ϵ range “Maximum” (1st quartile in range “Maximum”, Q1-max):
    P*_i_* ≥ P_*i*max_ − 1/4·(P_*i*max_ − P_0_) ⇒ n_max_ = ƩnP_i;_
  (2) *i* ϵ range “Minimum” (4th quartile in range “Minimum”, Q4-min):
    P*_i_* ≤ P_*i*min_ + 1/4·(P_0_ − P_*i*min_) ⇒ n_min_ = ƩnP*_i_*.
  *Note: mathematical description of other quartiles:
  - *i* ϵ range Q2-max: P_*i*max_ − 1/4·(P_*i*max_ − P_0_) > P*_i_* ≥ P_*i*max_ − 1/2·(P_*i*max_ − P_0_);
  - *i* ϵ range Q3-max: P_*i*max_ − 1/2·(P_*i*max_ − P_0_) > P*_i_* ≥ P_*i*max_ − 3/4·(P_*i*max_ − P_0_);
  - *i* ϵ range Q4-max: P_*i*max_ − 3/4·(P_*i*max_ − P_0_) > P*_i_* > P_0;_
  - *i* ϵ range Q1-min: P_*i*min_ + 3/4·(P_0_ − P_*i*min_) < P*_i_* < P_0;_
  - *i* ϵ range Q2-min: P_*i*min_ + 1/2·(P_0_ − P_*i*min_) < P*_i_* ≤ P_*i*min_ + 3/4·(P_0_ − P_*i*min_);
  - *i* ϵ range Q3-min: P_*i*min_ + 1/4·(P_0_ − P_*i*min_) < P*_i_* ≤ P_*i*min_ + 1/2·(P_0_ − P_*i*min_).
- Basov-Jimack algorithm (BJ-algorithm):
  (1) *i* ϵ range “Maximum” (BJ-max):
    - if P_*i*max_ − 1/10·(P_*i*max_ − P_*i*min_) ≥ P_0_ + 1/2·(P_*i*max_ − P_0_) and P_*i*max_ > P_0_ ≥ P_*i*min_ ≥ 0: P*_i_* ≥ P_*i*max_ − 1/10·(P_*i*max_ − P_*i*min_) ⇒ n_max_ = ƩnP_*i*_; or else:
    - if P_*i*max_ − 1/10·(P_*i*max_ − P_*i*min_) < P_0_ + 1/2·(P_*i*max_ − P_0_) and P_*i*max_ > P_0_ ≥ P_*i*min_ ≥ 0: P*_i_* ≥ P_*i*max_ − 1/4·(P_*i*max_ − P_0_) ⇒ n_max_ = ƩnP_*i*_;
  (2) *i* ϵ range “Minimum” (BJ-min):
    - if P_*i*min_ + 1/10·(P_*i*max_ − P_*i*min_) ≤ P_0_ − 1/2·(P_0_ − P_*i*min_), P_*i*max_ ≠ P_*i*min_, and ƩP*_i_*_ϵ Q2-Q4-min_ > 0: P*_i_* ≤ P_*i*min_ + 1/10·(P_*i*max_ − P_*i*min_) ⇒ n_min_ = ƩnP*_i_*; or else:
    - if P_*i*min_ + 1/10·(P_*i*max_ − P_*i*min_) > P_0_ − 1/2·(P_0_ − P_*i*min_), P_*i*max_ ≠ P_*i*min_, and ƩP*_i_*_ϵ Q2-Q4-min_ > 0: P*_i_* ≤ P_*i*min_ + 1/4·(P_0_ − P_*i*min_) ⇒ n_min_ = ƩnP*_i_*; or else:
    - if P_*i*max_ = P_*i*min_ = ƩP*_i_*_ϵ Q2-Q4-min_ = P*_i_* = 0: n_min_ = 0;
  where: n_min_ is nitrogen base pair numbers (*i*), which were included in the range “Minimum”; n_max_ is number of *i*, which was included in the range “Maximum”; if P*_i_* ≤ P_*i*min_ + 3/4·(P_0_ − P_*i*min_): *i* ϵ range from Q2-min to Q4-min (Q2-Q4-min).

The range of E^H^_cr_ was from 0.30 to 0.59, while the change in energy taken into account when calculating open states in the entire range was uniform and equaled to 0.01 (Table 2).

**Figure 3:**
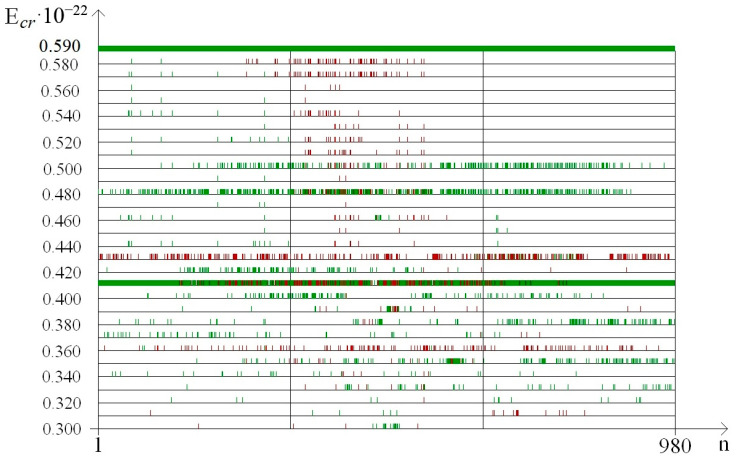
Distribution in the different parts of interferon alpha 17 (IFNA17) of nucleobase pairs (which was counted by decile method) leading after the single ^2^H/^1^H replacement the extreme frequency of open state (OS) occurrence. Note: red dot is the location of the deuterium atom in the DNA molecule, which leads to the maximum probability of OS occurrence (range); green dot is the location of the deuterium atom in the DNA molecule, which leads to the minimum probability of OS occurrence (range).

**Figure 4:**
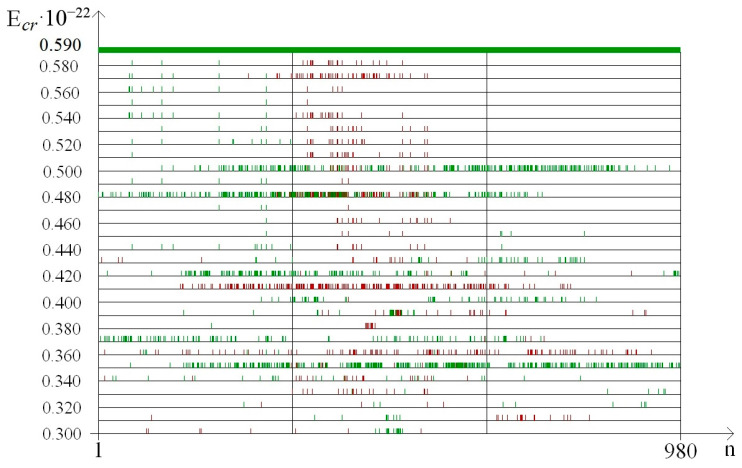
Distribution in the different parts of IFNA17 of nucleobase pairs (which was counted by quartile method) leading after the single ^2^H/^1^H replacement the extreme frequency of OS occurrence. Note: red dot is the location of the deuterium atom in the DNA molecule, which leads to the maximum probability of OS occurrence (range); green dot is the location of the deuterium atom in the DNA molecule, which leads to the minimum probability of OS occurrence (range).

**Figure 5:**
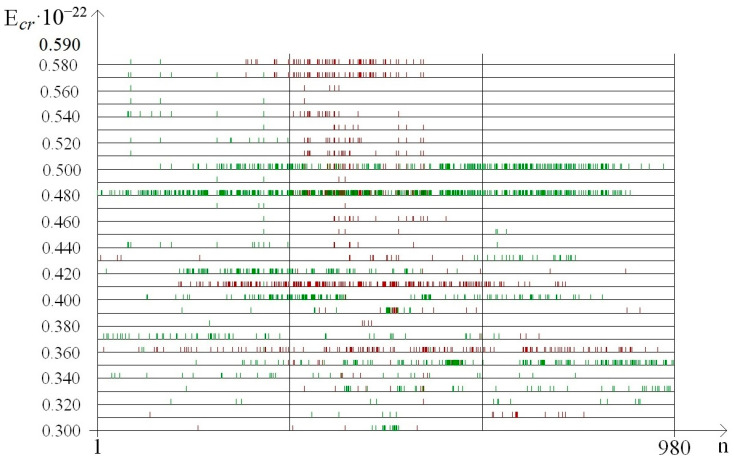
Distribution in the different parts of IFNA17 of nucleobase pairs (which was counted by Basov-Jimack algorithm) leading after the single ^2^H/^1^H replacement the extreme frequency of OS occurrence. Note: red dot is the location of the deuterium atom in the DNA molecule, which leads to the maximum probability of OS occurrence (range); green dot is the location of the deuterium atom in the DNA molecule, which leads to the minimum probability of OS occurrence (range).

In addition, to exact the number of n_min_ and n_max_ of the nitrogen base pairs in the range from 0.30 to 0.59 (where each E^H^_thr_ had n_min_ or n_max_ more than 0 for the whole gene), their number was also calculated for smaller ranges of E^H^_cr_:

- from 0.30 to 0.43, which were selected because of III part, which, in this range, had summary n_max_ more than 0; its n_max_ measured by decile method equaled 203 (Figure 3), quartile method equaled 88 (Figure 4), or BJ-algorithm equaled 87 (Figure 5);
- from 0.44 to 0.59 that were selected because of III part, which in this range had summary n_max_ equaled 0; its n_max_, measured by the each approach: decile method, quartile method, or BJ-algorithm, always equaled 0 (Figure 3).

After that, we used some statistical methods to exact the significance of differences among n_min_ and n_max_ of the three gene parts. Yates corrected chi-squared test (χ^2^_Yates_) was applied for a 2 × 2 contingency Table 3 (where degrees of freedom (ν) = (2–1)·(2–1) = 1). As a shortcut, for a 2 × 2 Table 3 with the following entries:

Where **A** and **B** are the rows according to the parts of the gene: I, II, or III; S is the column of n_min_ of the range “Minimum”, or n_max_ of the range “Maximum”; **F** is the column of the rest number of nucleotide pairs; *a* and *c* are n_min_ of the range “Minimum”, or n_max_ of the range “Maximum” in each compared part of the gene in the determined range of E^H^_thr_; *b* and *d* equals the total number of nucleotide pairs of the gene part minus n_min_ of the range “Minimum”, or n_max_ of the range “Maximum” in each compared part of the gene in the determined range of E^H^_thr_; *N*_A_ = *a* + *b*; *N*_B_ = *c* + *d*; *N*_S_ = *a* + *c*; *N*_F_ = *b* + *d*; *N* = *N*_A_ + *N*_B_ + *N*_S_ + *N*_F_.

χ^2^_Yates_ = *N*·(│*ad*–*bc*│–*N*/2)^2/^*N*_A_·*N*_B_·*N*_S_·*N*_F_.

Chi-square corrected by procedure for Bonferroni (χ^2^_B_) was used for a 3 × 2 contingency table (where 3 is rows, 2 is columns, ν = (3–1)·(2–1) = 2). The Kruskal–Wallis ANOVA by Ranks test (KWt) was applied for the comparison n_min_ of the range “Minimum”, or n_max_ of the range “Maximum” in the determined range of E^H^_cr_ for two gene parts that are mutually independent. Median test (Mt) was used for the comparison n_min_ of the range “Minimum”, or n_max_ of the range “Maximum” in the determined range of E^H^_cr_ for two gene parts that are mutually independent.

It was found out that the quantity of n_min_ and n_max_ of OSs for each gene part was different in the above-described ranges of E^H^_cr_ (Table 4). In the range of E^H^_cr_ from 0.30 to 0.59 the highest number of n_max_ was in the II part, e.g., when it was counted by the decile method (χ^2^_B_: *p* < 0.001) the n_max_ in the II part was in 2.76 and 2.89 times higher than in the I part and III part respectively, but for the quartile method (χ^2^_B_: *p* < 0.001) the n_max_ in the II part was in 5.35 and 6.69 times higher, and according to BJ-algorithm (χ^2^_B_: *p* < 0.001) the n_max_ in the II part was in 4.74 and 5.94 times higher compared to the I part and III part respectively (Table 4). Wherein in the same range of E^H^_cr_ in the II part of IFNA17, the highest number of n_min_ also was only if it was counted by BJ-algorithm (χ^2^_B_: *p* < 0.002), which showed that the n_min_ in the II part was in 1.22 and 1.30 times higher than in the I part and III part, respectively. Whereas the quantity of n_min_ calculated by the quartile method turned higher in the I part compared to the II part and III part by 9.2% and 48.5% respectively (χ^2^_B_: *p* < 0.001, Table 4). It should be noted, that n_min_, counted by the decile method at the same condition, had no differences in all parts of the gene (χ^2^_B_: *p* > 0.2).

Moreover, the comparison of the total n_max_ number of the gene ends (sum of I part and III part) to the base pair amount of the range “Maximum” in the center gene part revealed that the II part had more n_max_ than the both ends of IFNA17; which was 1.41 times more by the decile method (pχ^2^_Yates_ < 0.0001), 2.97 times more by the quartile method (pχ^2^_Yates_ < 0.0001), and 2.64 times more by BJ-algorithm (pχ^2^_Yates_ < 0.0001). In contrast, the nucleobase quantities of the range “Minimum” had no difference between ends and center part of gene calculated by decile method (pχ^2^_Yates_ = 0.057), but n_min_ difference counted by quartile method was significant (pχ^2^_Yates_ = 0.012), and higher than the difference by BJ-algorithm (pχ^2^_Yates_ = 0.0001).

The comparing of nucleobase quantities of the range “Maximum” depending on each E^H^_cr_ (from 0.30 to 0.59) in the different parts of gene showed, that there was the difference among them, and if it was counted by the decile method, by the quartile method, or by BJ-algorithm then the p_KWt_ and p_Mt_ always were less than 0.0001 (Figure 3, Figure 4 and Figure 5). In contrast of it the nucleobase quantities of the range “Minimum” had no difference when counting by the decile method (p_KWt_ = 0.223; p_Mt_ = 0.325, Figure 3), but the difference was found for n_min_ computed by the quartile method (p_KWt_ = 0.046; p_Mt_ = 0.079, Figure 4) and by BJ-algorithm (p_KWt_ = 0.034; p_Mt_ = 0.111, Figure 5). Additionally, imparity between n_max_ of the ends of IFNA17 and base pair quantity of the range “Maximum” in its center was also proved by the decile method, quartile method, and BJ-algorithm (p_KWt_ < 0.001; p_Mt_ < 0.001 in all cases). However, it did not achieve any significant p_KWt_ or p_Mt_ by all approaches to the n_min_, and each p_KWt_ and p_Mt_ was more than 0.12 in this range of E^H^_cr_.

At the same time in the range of the higher E^H^_cr_ (from 0.44 to 0.59), the biggest base pair quantity of the range “Maximum” was also in the II part (χ^2^_B_: *p* < 0.001), e.g., that n_max_ number calculated by the each method in the II part of the gene was more than 15.5 times higher than in its I part (pχ^2^_Yates_ < 0.0001, Table 4). Moreover, for the III part of IFNA17, there were no nucleotides in the range “Maximum” at all for these energies, so n_max_ of the center part of gene was also more than 15.5 times higher compared to n_max_ of its ends (pχ^2^_Yates_ < 0.0001). Comparison of n_max_ in the three parts of gene, depending on the each E^H^_cr_ from 0.44 to 0.59, pointed out to the significant difference among them, and if it was counted by the decile method, by the quartile method, or by BJ-algorithm then the p_KWt_ and p_Mt_ always were less than 0.0001 (Figure 3, Figure 4 and Figure 5). In contradistinction to this, if n_min_ was counted by decile method (χ^2^_B_: *p* > 0.5), and by BJ-algorithm (χ^2^_B_: *p* > 0.2) the nucleobase quantities in the range “Minimum” had no differences among three parts of IFNA17, but the difference was found by the quartile method (χ^2^_B_: *p* < 0.002, Table 4). In addition, there was no statistical inequality of n_min_ between II part and III part (pχ^2^_Yates_ from 0.0816 to 0.1947 dependent on the counting method), ends and center of the gene (pχ^2^_Yates_ from 0.1699 to 0.3313 dependent on the counting method), but if it was computed by the quartile method the base pair amount in the range “Minimum” of the I part was higher compared to the II part and III part by 16.9% (pχ^2^_Yates_ = 0.0099) and 27.1% (pχ^2^_Yates_ = 0.0001) respectively (Table 4). Moreover, when comparing n_min_ in the I, II, and III parts of IFNA17, depending on the each E^H^_cr_ from 0.44 to 0.59, the significant difference among them was found, which has p_KWt_ = 0.0034 and p_Mt_ = 0.0080 if it was counted by the decile method, p_KWt_ = 0.0006 and p_Mt_ < 0.0001—by the quartile method, and also p_KWt_ = 0.0002 and p_Mt_ < 0.0001–by BJ-algorithm. The comparison of the ends and center of the gene, depending on the each E^H^_cr_ in this energy range, revealed significant differences between both n_max_ and n_min_ by all calculating methods (for n_max_: p_KWt_ were less than 0.0001, and p_Mt_–from less than 0.0001 to 0.0010; for n_min_: p_KWt_ were from 0.0003 to 0.0060, and p_Mt_–from 0.0012 to 0.0131).

In the range of E^H^_cr_ from 0.30 to 0.43 according to the decile method (χ^2^_B_: *p* < 0.001, Figure 3) in the II part of IFNA17, the total nucleotide number of the range “Maximum” was higher than n_max_ of the I part in 1.65 times (pχ^2^_Yates_ < 0.0001) and n_max_ of the III part in 1.58 times (pχ^2^_Yates_ < 0.0001). The quartile method demonstrated (χ^2^_B_: *p* < 0.001, Figure 4) that in this energy range the II gene part had the total number of n_max_ significant higher compared to n_max_ of the I part (in 3.10 times, pχ^2^_Yates_ < 0.0001), or it of the III part (in 3.24 times, pχ^2^_Yates_ < 0.0001). According to BJ-algorithm (χ^2^_B_: *p* < 0.001, Figure 5), the similar number in the II part of IFNA17 was higher than n_max_ of the I part in 2.74 times (pχ^2^_Yates_ < 0.0001) and n_max_ of the III part in 2.89 times (pχ^2^_Yates_ < 0.0001, Table 4). The comparison of the base pair amounts of the range “Minimum” among three parts of the gene showed differences by the decile method (χ^2^_B_: *p* < 0.002), quartile method (χ^2^_B_: *p* < 0.001), and BJ-algorithm (χ^2^_B_: *p* < 0.001). Moreover, gene part, included the highest n_min_, was different depending on the counting method, e.g., if it was calculated by the decile method the III part had the biggest amount of n_min_: by 25.3% and 23.9% more than the I part (pχ^2^_Yates_ < 0.0002) and II part (pχ^2^_Yates_ < 0.0005) respectively; and if it was computed by the quartile method then the I part had the highest n_min_: by 79.4% more than the III part (pχ^2^_Yates_ < 0.0001), but not the II part (pχ^2^_Yates_ = 0.74); and if it was counted by BJ-algorithm the II part had the biggest amount of n_min_: by 45.1% more than the both I part and III part (pχ^2^_Yates_ = 0.0002, Table 4). In addition, n_max_ in the I, II, and III part of IFNA17 depending on the each E^H^_cr_ (from 0.30 to 0.43) were frequently unequal, such as these counted by the decile method (p_KWt_ = 0.0493, p_Mt_ = 0.0764, Figure 3), by the quartile method (p_KWt_ = 0.0335, p_Mt_ = 0.0244, Figure 4), or by BJ-algorithm (p_KWt_ = 0.0220, p_Mt_ = 0.0293, Figure 5); but the base pair amounts of the range “Minimum” dependent on similar conditions in these gene parts had always no differences, e.g., calculated by the decile method (p_KWt_ = 0.491, p_Mt_ = 0.424), by the quartile method (p_KWt_ = 0.386, p_Mt_ = 0.135), or by BJ-algorithm (p_KWt_ = 0.308, p_Mt_ = 0.215).

The difference of the nucleobase quantities of the range “Maximum” between ends and center of the gene was much expressed: pχ^2^_Yates_ was less 0.0001 if n_max_ was counted by the decile method, the quartile method, or BJ-algorithm. Moreover, comparison of the gene ends to the gene center (dependent on the each E^H^_cr_), revealed that the p_KWt_ were less than 0.05 for n_max_, calculated by the quartile method (0.0475) or BJ-algorithm (0.0295), and p_Mt_ were less than 0.05 for n_max_, counted by the decile method and BJ-algorithm (both equaled 0.0233). On the other side in this energy range (dependent on the each E^H^_cr_) n_min_ in the ends of IFNA17 comparing to its amount in the center part of the gene had no difference by the decile method (p_KWt_ = 0.505, p_Mt_ = 0.449), by the quartile method (p_KWt_ = 0.382, p_Mt_ = 0.449), or by BJ-algorithm (p_KWt_ = 0.420, p_Mt_ = 0.449).

It should be also noted, that among three gene parts (out of dependent on the energy range), the differences between the total amount of n_max_ and n_min_ were the least in the II part of gene: from 38.3% (range of E^H^_cr_: 0.30–0.43, n_min_ > n_max_) to 115.1% (range of E^H^_cr_: 0.44–0.59, n_min_ > n_max_) if counted by the decile method, from 59.8% (range of E^H^_thr_: 0.44–0.59, n_min_ > n_max_) to 90.5% (range of E^H^_cr_: 0.30–0.43, n_min_ > n_max_)–by the quartile method, or from 7.2% (range of E^H^_cr_: 0.30–0.43, n_min_ < n_max_) to 11.8% (range of E^H^_cr_: 0.44–0.59, n_min_ < n_max_)–by BJ-algorithm. In contrast of this, the higher differences between the total amount of n_max_ and n_min_ were more often in the III part of gene counted by the decile method, or in the I part–by the quartile method (Table 4).

## 4. Discussion

Albeit there should be no difference theoretically in three parts (I, II, and III) of IFNA17 between the amounts of the n_min_ or n_max_ of OSs dependent on the single ^2^H/^1^H replacement, because each part had actually the same number of nitrogenous base pairs in the double-stranded DNA forming it [36], the mathematical modeling demonstrated much more significant differences between their amounts in the described gene parts. The appearance in any gene part of some significant fluctuation of the amount of nucleotide pairs both with the higher P*_i_* and the lower P*_i_* compared to the P_0_ (which correlates to the essential OS frequency) leads to the higher risk of changing the whole DNA functional activity [37]. It is possible due to the fact that a stability of double-stranded DNA supported by the H-bonding between complementary nitrogenous bases and the stacking formed with neighboring bases [38], so the fluctuation opening even of one nucleobase in duplex DNA can involve disruption of the whole stacking in the determined gene part [37,39]. Moreover, decrease of P*_i_* in the any gene part lower than essential P_0_ level can be also a strong negative factor, because the spontaneous base flipping is the necessary part of some catalyzed processes involving DNA [40,41]. So, for the catalytic activity of DNA glycosylase, 8-oxoguanine DNA glycosylase I, and alkyl adenine DNA glycosylase (which are the important DNA repair enzymes [42]), as well as DNA methyltransferase family enzymes and β-glucosyl transferase (which provide selective modification of DNA [43]) the natural base-flipping frequency needs to be determined. All the mentioned enzymes interact with DNA only according to the nucleobase completely flipped out of the helix structure due to the previous occurrence of OSs in the specific gene region [44,45,46,47]. In addition, the rate of the transcription bubble passages is also dependent on the essential frequency of the open states in the selective part of gene [27,48,49,50]. Also, both slowdown and rev up of molecular dynamic of DNA due to the change of occurrence OS rate play the significant role in the impairment of DNA-mediated charge transfer (CH–π interaction), which can lead to the dysfunctional activity of repair enzymes [51,52,53], and, consequently, to the increase rate of mutations.

**Figure 6:**
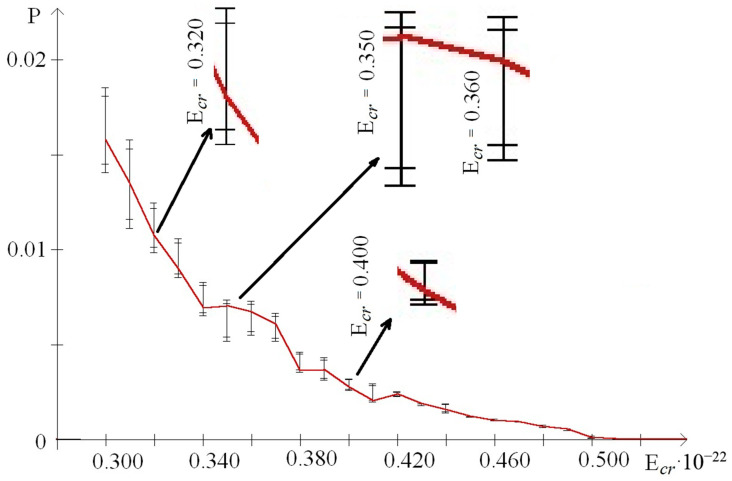
Dynamics of the open state (OS) occurrence in IFNA17 dependent on the H-bond dissociation energy under natural condition and after the single ^2^H/^1^H replacement (with gradation of OS occurrence frequency by decile method). Note: for each H-bond dissociation energy: the 1st cross dash is P_*i*max_, the 2nd cross dash is bottom of the 10th decile, the 3nd cross dash is top of the 1st decile; the 4th cross dash is P_*i*min_; red line is OS occurrence frequency under condition when all hydrogen bonds in DNA are ^1^H (P_0_).

**Figure 7:**
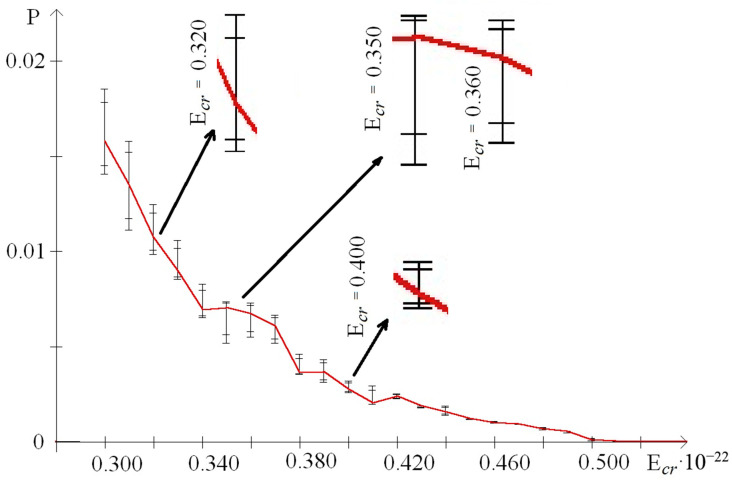
Dynamics of the open state (OS) occurrence in IFNA17 dependent on the H-bond dissociation energy under natural condition and after the single ^2^H/^1^H replacement (with gradation of OS occurrence frequency by quartile method). Note: for each H-bond dissociation energy: the 1st cross dash is P_*i*max_, the 2nd cross dash is the bottom of Q1-max,, the 3rd cross dash is the top of Q4-min; the 4th cross dash is P_*i*min_; red line is OS occurrence frequency under condition when all hydrogen bonds in DNA are ^1^H (P_0_).

**Figure 8:**
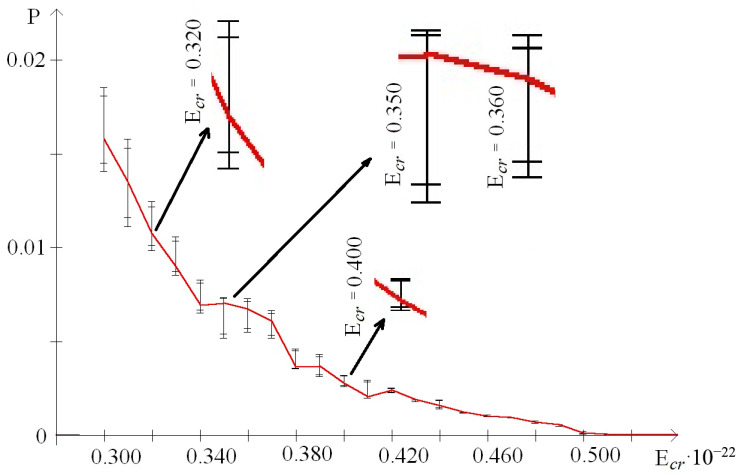
Dynamics of the open state (OS) occurrence in IFNA17 dependent on the H-bond dissociation energy under natural condition and after the single ^2^H/^1^H replacement (with gradation of OS occurrence frequency by Basov–Jimack algorithm). Note: for each H-bond dissociation energy: the 1st cross dash is P_*i*max_, the 2nd cross dash is bottom of the range “Maximum”, the 3rd cross dash is top of the range “Minimum”; the 4th cross dash is P_*i*min_; red line is OS occurrence frequency under condition when all hydrogen bonds in DNA are ^1^H (P_0_).

In our study the unequal sensitivity of the different parts of IFNA17 to the single ^2^H/^1^H replacement was demonstrated, which is manifested in the differently expressed changes of their OS frequency. Among three above-described counting approaches of the nucleobase amounts, which can be included in the range “Maximum” or “Minimum” based on the OS frequency occurring after the single ^2^H/^1^H replacement at the specified DNA base pair, the BJ-algorithm should be highlighted. That conclusion can be explained due to the least false positive and false negative results if we are using this algorithm compared to the decile or the quartile methods for the nucleobase counting. For example, in the I and III parts of IFNA17 in the range of E^H^_cr_ from 0.30 to 0.43, the base pair amounts of “Minimum” calculated according to the decile and quartile methods were different (pχ^2^_Yates_ ≤ 0.0002), but it was because of numerous of the false positive results in data counted by the decile method at E^H^_cr_: 0.38 (n_min_ were 13 and 80) and 0.41 (n_min_ were 266 and 308 in the I and III parts respectively, Figure 3), and by the quartile method at E^H^_cr_: 0.35 (n_min_ were 283 and 202), 0.36 (n_min_ were 26 and 1), 0.37 (n_min_ were 154 and 36), 0.42 (n_min_ were 66 and 8), and 0.43 (n_min_ were 3 and 25 in the I and III parts respectively, Figure 4). It was observed due to the shifts of P_0_ between extreme values of P*_i_*: closer to P_min_ or to P_max_ dependent on E^H^_cr_ (Figure 6 and Figure 7), that produced the false positive and false negative results in the calculations. In contrast to these methods, BJ-algorithm took into account the mentioned shifts of P_0_ (Figure 8), that actually avoided the false results and revealed no difference between I and III parts of the gene in this energy range (pχ^2^_Yates_ = 0.954).

On the other side, such a new approach cutting down false positive results provides the possibility to find out the significant differences between frequency of OSs in IFNA17 due to the single ^2^H/^1^H replacement in the highlighted gene parts. Although, in the range of E^H^_cr_ from 0.30 to 0.43 in the I and II gene parts the nucleobase amounts of “Minimum” computed by the decile method had no statistical difference (pχ^2^_Yates_ = 0.85) like the similar nucleobase amounts of the same gene parts calculated by the quartile method (pχ^2^_Yates_ = 0.74), the n_min_, which were by BJ-algorithm, had reliable difference (pχ^2^_Yates_ = 0.0002), because the last approach diminished total base pair amounts due to the taking away of false positive results: at E^H^_cr_ 0.38 and 0.41 it was 277 and 209 false n_min_ less than calculated by the decile method (Figure 3); at E^H^_cr_ 0.35–0.37 and 0.42–0.43 it was 424 and 347 false n_min_ less than–by the quartile method in I and II parts respectively (Figure 4). However, with that, the new algorithm allowed to decrease false negative results, e.g., when counting n_min_ in the ends of gene and its center part at E^H^_cr_ 0.32–0.33 and 0.40 (compared to the quartile method, Figure 4 and Figure 5), which increases the reliability of the approach. Another example of the appropriateness of using BJ-algorithm for processing study data is the finding of difference between n_min_ of the ends and center of IFNA17 (pχ^2^_Yates_ < 0.0001), in contrast to the usage of the decile method (pχ^2^_Yates_ = 0.565).

Moreover, more efficient and sustainable usage of BJ-algorithm for identifying n_max_ in the I, II and III parts of IFNA17 confirmed the lowest *p*-values (p_KWt_ = 0.0220, p_Mt_ = 0.0293) compared to the similar *p*-values found for n_max_, which were filtered by the decile and quartile methods (as the pure fractile approach according to the properties of the pure function [54]). Moreover, the *p*-values of n_max_ between ends and center of the gene were both p_KWt_ and p_Mt_ less than 0.05 (0.0295 and 0.0233, respectively) if they were counted only by BJ-algorithm. The biggest amount of false positive results of n_max_ calculated by the decile method was at E^H^_cr_ 0.43 (Figure 3), the biggest amount of false negative results of n_max_ filtered by the quartile method was at E^H^_cr_ 0.36 (Figure 4).

In the range of E^H^_cr_ from 0.44 to 0.59 the difference among data filtered by three methods was also significant, and BJ-algorithm showed higher efficacy for processing study numbers of OS frequency. For example, the amount of n_max_ in the gene center part filtered by the new algorithm was on 39 n_i_ (which can be considered as false positive results according to the robust estimation) less than n_max_ counted by the quartile method. However, more expressed differences were between n_min_ (Table 4), that explained due to the convergence of P_*i*max_ and P_*i*min_, also P_0_ and 0 in the range of higher energies (Figure 8). For example, the decile method leads to many false positive results of n_min_ at E^H^_cr_ 0.46 and 0.59, also quartile method–at E^H^_cr_ 0.55 and 0.59, that provoked among n_min_ of three gene parts (each of which had the same own P*_i_* data) completely different *p*-values: χ^2^_B_: *p* > 0.5 (decile method), χ^2^_B_: *p* < 0.002 (quartile method), and χ^2^_B_: *p* > 0.2 (BJ-algorithm).

Contrary to the lower range of E^H^_cr_ when the fractile methods produced both false positive and false negative results of extreme nucleobase amounts (both n_max_ and n_min_), in the higher range of E^H^_cr_ they much more frequently cause be false positive results in range “Minimum”, that stemmed from the following properties of DNA OS frequency: P_*i*max_ → P_*i*min_ and P_0_ → 0, so all of these *p* inversely correlated to E^H^_cr_ (Table 2). In general, all these practical examples proved the conclusion about the possibility of using BJ-algorithm as the more effective filtering approach of described study data compared to the pure fractile methods. Regarding the above-mentioned information, data will be further considered as filtered only by BJ-algorithm.

Nevertheless, despite the same length of the I, II, and III parts of IFNA17, the study data filtered by each of the methods pointed out to the statistically significant differences of n_max_ and n_min_ of the gene ends and its center, which confirmed heterogeneous sensitivity at the considered gene regions to the single ^2^H/^1^H replacement. It is noteworthy that in the range of E^H^_cr_ from 0.30 to 0.43 the center part of the gene is the most prone to both fluctuations of n_max_ (more than in 2.73 times) and n_min_ (in 1.45 times) compared to the I and III gene parts. Wherein in the higher energy range (0.44–0.59) II part of IFNA17 significant different from I and III its parts by nucleobase pair amount only in the range “Maximum” (more than in 15.58 times), but there was no difference between n_min_, because of the convergence extremes of OS frequency in DNA.

All described fluctuations of n_max_ and n_min_ after single ^2^H/^1^H replacement can result in DNA damage due to the slowdown in the rate of bubble forming (especially non-superhelical stress-induced denaturation bubbles having more than 4 base-pairs), including the promoter regions and binding points of specific proteins (e.g., DNA repair enzymes [41,55]), or because of the overwhelming rate of flipping-out DNA nucleobases that can be accompanied by increasing both the speed of modification of their chemical structure and mismatched protein-DNA interaction [56,57,58,59].

Our data clearly demonstrate the possibility of higher risk of gene lesion in all its length due to single ^2^H/^1^H replacement predominantly in the center part of IFNA17 in all studied critical energies (from 0.30 to 0.59), which show abnormal rate of occurring OSs, that manifested more often its increasing (by 24%) compared to decreasing. According to the occurrence rate of P_*i*max_ in the range of E^H^_cr_ from 0.44 to 0.59, the single deuterium introduction in the III part of gene actually did not cause any changes compared to the natural frequency of OSs as opposed to the single ^2^H/^1^H replacement in I or II parts. So, after deuterium introduction in the III part, predominated n_min_, which can lead to the sharp slowdown of DNA bubble forming, which has more than 4 nucleobases participating in generation of the preexisting state of denaturation, that is required by specific DNA-binding enzymes [56,59], and can be a predictor of function lesion of DNA repair system.

It was well known that different DNA loci have inequality in the adenine-thymine/guanine-cytosine (A-T/G-C) ratio. In IFNA17 I part has 163 A-T and 164 G-C base pairs, II part has 186 A-T and 140 G-C base pairs, and III part has 236 A-T and 91 G-C base pairs [30]. Despite the fact, that I part has the minimum number of A-T pairs and the III part has the maximum number of A-T pairs (compared to each other), the most significant change of OS frequency in IFNA17 was found out after single ^2^H/^1^H replacement in the II part of gene (which contained intermediate AT percentage) in the all studied energy ranges (it was observed under both E^H^_cr_ diapasons: from 0.30 to 0.43 and from 0.44 to 0.59, Table 4). All of this definitely points out that not only total number (or percentage) of AT nucleobases influences the OS frequency [60], but the specific sequence of A-T and G-C bases, or DNA pattern, which can lead to the both increase and decrease of OS amount in the gene.

Thus, all of described cases of dynamics of the double-stranded DNA confirmed the heterogeneous sensitivity of IFNA17 to the ^2^H/^1^H exchange dependent on an energy of external influences on the gene. The study also showed that the single deuterium introduction in IFNA17 even in the III gene part, which is out of the coding region (from 50th to 619th nucleobases [61,62]), can influence the transcription speed by modifying the dynamics of a double-stranded DNA due to the decreasing of OS amounts in other gene parts. Moreover, the coding region of IFNA17, which is more relevant to I and II parts, is the most sensitive part of the gene by the single ^2^H/^1^H replacement. However, our study had some limitations. One of them was that all results have been obtained in the frameworks of the mathematical model based on the Newton’s equations and representative of the Cauchy problem for the system of 2n ordinary differential equations, which do not entirely take into account the contribution of DNA-protein interactions. The second limitation is increasing data variability in the low energy range (less than 0.30) due to the properties of the system of 2n ordinary differential equations. Nevertheless, we do not consider these limitations as an insurmountable obstacle, and think that the approach can be generalized and used to more complex models of DNA dynamics.

## 5. Conclusions

In general, the mathematical modeling study demonstrated significant inequality (dependent on single ^2^H/^1^H replacement in DNA) among three parts of gene similar in length of the frequency of occurrence of the OSs both with the higher P*_i_* and the lower P*_i_* compared to the natural P_0_. The bases pairs with P*_i_* significantly higher than P_0_ can provide the bigger risk of mutation (due to a change in the ratio of the equilibrium constants of the denaturation bubble opening reaction in various DNA parts, including the promoter regions, and the thermodynamic indices at binding points of specific enzymes [27,63,64]), whereas the nucleotide pairs with Pi significantly lower than P_0_ can lead to the harsh slowdown of transcription or replication compared to the natural level of failures in those essential processes [49,50,55]. In addition, in the study it was found out that through the range of H-bond dissociation energy more than 0.58 and approximated to 0.59 the DNA OSs were not occurred in IFNA17, and only close states of nitrogenous base pairs were revealed in the whole gene.

In this paper the new convenient approach of the analysis of the abnormal frequency of OSs in the different part of IFNA17 was presented, which took into account both rising and decreasing of them that allows to make a prediction of the functional instability of the specific DNA regions. One advantage of the new algorithm is diminishing the number of both false positive and false negative results in data filtered by this approach compared to the pure fractile methods, such as deciles or quartiles. Thus, study data pointed out to the statistically significant differences of nucleobase pair amounts, which can be included in the extreme ranges (“Maximum” or “Minimum”) based on the frequency of OS occurrence, in the gene ends and its center, so the dynamics of a double-stranded DNA confirmed its heterogeneous sensitivity to the single ^2^H/^1^H replacement dependent on energy of external influences on the gene. It was also shown that the single deuterium exchange in IFNA17 even out of the coding region (from 50th to 619th base pairs), e.g., in the III gene part, can probably influence the transcription speed by modifying the dynamics of a double-stranded DNA due to the decreasing of OS amounts in other gene parts.……

## Figures and Tables

**Table 1:** Equation coefficients (1′)–(2‴).

Type of Base	A	T	G	C
$I\cdot 10^{-44},\;\mathrm{kg}\cdot m^{2}$	7.61	4.86	8.22	4.11
*R*, Å	5.80	4.80	5.70	4.70
$K\cdot 10^{-18}$, J	2.35	1.61	2.27	1.54
$k_{12}^{H}\cdot 10^{-2},\;N/m$	6.20	6.20	9.60	9.60
$\beta \cdot 10^{-34},\;J\cdot s$	4.25	2.91	4.10	2.79

**Table 2:** Probabilities of occurrence of open states between different nitrogenous bases in double-stranded DNA dependent on single ^2^H/^1^H replacement

$E_{cr}^{H}\cdot 10^{-22}$ N·m	P_0_	*i*_min_, or nCS	*i*_max_	$P_{i_{min}}$	$P_{i_{max}}$
0.30	0.0158002	498	544	0.0140623	0.0185293
0.31	0.0135005	365	711	0.0111363	0.0157753
0.32	0.0107263	475	555	0.0098598	0.0124513
0.33	0.0089972	858	405	0.0085322	0.0105687
0.34	0.0069408	299	435	0.0065120	0.0082811
0.35	0.0070181	512	383	0,0051732	0.0073687
0.36	0.0067221	80	459	0.0054978	0.0072946
0.37	0.0060742	102	651	0.0051911	0.0066520
0.38	0.0036320	191	451	0.0035676	0.0046222
0.39	0.0036706	553	506	0.0031209	0.0043160
0.40	0.0027459	688	422	0.0026089	0.0032148
0.41	0.0020461	284	487	0.0019833	0.0029466
0.42	0.0024118	526	341	0.0022690	0.0025324
0.43	0.0018781	676	509	0.0018024	0.0018882
0.44	0.0015812	284	429	0.0014226	0.0018898
0.45	0.0012137	678	422	0.0011902	0.0012356
0.46	0.0010036	284	422	0.0009866	0.0010921
0.47	0.0009419	284	422	0.0009282	0.0009590
0.48	0.0006775	284	422	0.0006355	0.0007785
0.49	0.0005409	284	422	0.0004716	0.0005861
0.50	0.0001030	284	422	0.0000739	0.0002028
0.51	0.0000386	58	422	0.0000297	0.0000598
0.52	0.0000279	284	395, 403, 404, 411,422, 513	0.0000273	0.0000284
0.53	0.0000252	284	411, 422	0.0000243	0.0000261
0.54	0.0000151	52, 53, 58, 73, 93,108, 127, 204, 284	334, 345, 353, 358, 359, 361, 362, 376, 378, 383, 388, 389, 392, 395, 403,404, 411, 444, 513	0.0000148	0.0000153
0.55	0.0000142	58, 108, 284	353	0.0000138	0.0000145
0.56	0.0000132	58	353, 395, 404, 411	0.0000127	0.0000135
0.57	0.0000120	58	353, 395, 403, 404, 411	0.0000114	0.0000124
0.58	0.0000104	58	353	0.0000090	0.0000111
0.59	0.0000000	nCS = 980	-	0.0000000	-

Note: CS is close state (P_CS_ = 0).

**Table 3:** Chi-squared test with Yates correction.

	S	F	
**A**	*a*	*b*	*N*_A_
**B**	*c*	*d*	*N*_B_
	*N*_S_	*N*_F_	*N*

**Table 4:** Quantity of the n_min_ and n_max_ in double-stranded DNA dependent on single ^2^H/^1^H replacement was counted by different approaches in some ranges of the E^H^_cr._

$\mathrm{Range}\;\mathrm{of}\;E_{cr}^{H}\cdot 10^{-22}$ N·m, n	Part of IFNA17	Approach		
		Decile Method	Quartile Method	BJ-Algorithm
0.30–0.59 n_max_	I	212	110	109
	II	586 #	589 #	517 #
	III	203 ¤	88 ¤	87 ¤
0.30–0.59 n_min_	I	998	1124	386
	II	1014	1029 *	472 #
	III	1080	757 #, ¤	363 ¤
0.30–0.43 n_max_	I	195	92	92
	II	321 #	285 #	252 #
	III	203 ¤	88 ¤	87 ¤
0.30–0.43 n_min_	I	439	556	162
	II	444	543	235 #
	III	550 #, ¤	310 #, ¤	162 ¤
0.44–0.59 n_max_	I	17	18	17
	II	265 #	304 #	265 #
	III	0 #, ¤	0 #, ¤	0 #, ¤
0.44–0.59 n_min_	I	559	568	224
	II	570	486 #	237
	III	530	447 #	201

Note: * is *p*-value (χ^2^_Yates_) < 0.05 compared to the similar n of the I part, measured by the same method; # is *p*-value (χ^2^_Yates_) < 0.01 compared to the similar n of the I part, measured by the same method; ¤ is *p*-value (χ^2^_Yates_) < 0.01 compared to the similar n of the II part, measured by the same method.
